# Lifestyle Behaviors and Quality of Life Among Older Adults After the First Wave of the COVID-19 Pandemic in Hubei China

**DOI:** 10.3389/fpubh.2021.744514

**Published:** 2021-12-10

**Authors:** Yanping Duan, D. L. I. H. K. Peiris, Min Yang, Wei Liang, Julien Steven Baker, Chun Hu, Borui Shang

**Affiliations:** ^1^Department of Sport, Physical Education and Health, Faculty of Social Sciences, Hong Kong Baptist University, Kowloon Tong, Hong Kong SAR, China; ^2^Centre for Health and Exercise Science Research, Hong Kong Baptist University, Kowloon Tong, Hong Kong SAR, China; ^3^College of Health Sciences, Wuhan Institute of Physical Education, Wuhan, China; ^4^Student Mental Health Education Center, Northwestern Polytechnical University, Xian, China; ^5^Department of Social Science, Hebei Sport University, Shijiazhuang, China

**Keywords:** physical activity, fruit and vegetable intake, preventive behaviors, quality of life, socioeconomic status, older adults, COVID-19 pandemic

## Abstract

**Background:** Older adult quality of life (QoL) is facing huge challenges during the COVID-19 pandemic. New normal lifestyle behaviors, including getting adequate physical activity (PA), consuming sufficient fruits and vegetables (FV) and enacting individual preventive behaviors (frequent hand washing, facemask wearing, and social distancing), as a significant determinant for QoL, have not been adequately addressed in older adults during the pandemic. This study aimed to investigate the characteristics of QoL in Chinese older adults after the first wave of the COVID-19 pandemic in Hubei China. The objective of the study was to examine any associations of lifestyle behaviors with QoL, and to identify the moderating role of socioeconomic indicators in the associations identified.

**Methods:** A cross-sectional study was conducted in Hubei, China, from June 15, 2020, to July 10, 2020. Five hundred sixteen older adults completed an online survey (mean age = 67.6 ± 6.6; 57.9% women). The questionnaire consisted of demographic information, covariates (chronic diseases and infected cases of acquaintances), lifestyle behaviors [PA stage, FV intake (FVI) stage and three preventive behaviors], and QoL. *T*-tests, ANOVA tests, multiple linear regression models with simple slope analyses were used to test the hypotheses.

**Results:** QoL significantly differed in relation to economic situation, chronic diseases, marital status, education, living situation, age group, and professional status. Participants' economic situation (*β*_average vs. below average_ = 0.17, *p* < 0.01; *β*_above average vs. below average_ = 0.15, *p* < 0.01), chronic diseases (*β*_yes vs. no_ = 0.19, *p* < 0.001), FVI stage (*β* = 0.21, *p* < 0.001), and preventive behaviors (*β* = 0.10, *p* < 0.05) indicated a significant association with QoL. Education level and economic situation significantly interacted with preventive behaviors on QoL, respectively (*β*_preventive behaviors × *educational level*_ = −1.3, *p* < 0.01; *β*_preventive behaviors × *economic situation*_ = −0.97, *p* < 0.05).

**Conclusions:** Findings emphasize the importance of enhancing FVI and preventive behaviors on QoL improvement in older adults during the COVID-19 pandemic. Older adults who are in a lower economic situation with lower education levels should be given priority when implementing interventions to improve preventive behaviors and QoL in older adults.

## Background

The novel coronavirus disease (COVID-19), a global health emergency and worldwide threat, contributed to over 161 million confirmed cases and over 3 million deaths worldwide as of 20th July 2021, including 119,784 confirmed cases and 5,617 deaths in China (1). Considerable evidence demonstrates that the likelihood of suffering from severe illness and death related to COVID-19 increases with age (2). Older adults (60 years old and above) are one of the most susceptible and vulnerable populations for being infected with COVID-19 (3).

During the COVID-19 pandemic, healthy aging advocacy is facing a big challenge. Maintaining a relatively high quality of life (QoL) in the elderly is an important indicator of healthy aging. QoL is considered, in general, a broad-ranging concept affected in a complex way by physical health, psychological state, personal beliefs, individual social relationships, and their relationships with the environment (4). A recent systematic review indicated that individuals' quality of life worsened during the COVID-19 pandemic and was more serious for older adults (5). Thus, it is crucial to identify and understand the factors contributing to a good QoL among older adults during the pandemic.

Many studies have indicated that healthy lifestyle behaviors relevant to health promotion and disease prevention present a considerable contributor to improved quality of life and lower morbidity and mortality among older adults (6–9). Performing adequate physical activity and consuming sufficient fruit and vegetables have been identified as two important health promotion behaviors because of their effective roles in improving physical and mental health in older adults (10–14). However, self-isolation and restrictions during the pandemic dramatically reduced the opportunities for the public to be physically active (15). In addition, there has been a high prevalence of unhealthy diets (e.g., insufficient fruit and vegetable intake) during the pandemic (16). These behavior changes may lead to negative health consequences and a low level of QoL among older adults (17).

Also, during the COVID-19 pandemic, individual disease preventive behaviors, including frequent hand washing, facemask wearing, and social distancing in public areas, play an important role in reducing the transmission of COVID-19 in the community (18). Because there is still not enough vaccination prevention for COVID-19 worldwide and in anticipation of rapidly mutating viruses which transitions may not be prevented by vaccinations, performing individual preventive behaviors in daily life, as a new healthy lifestyle behavior, will be paramount in preventing the spread of the virus. A recent study indicated that preventive behaviors could directly affect the QoL among the general population (19). As older adults are at a higher risk of infection of COVID-19, investigating the impact of preventive behaviors on QoL in older adults should be prioritized. To the best of our knowledge, few studies have examined the relationship between all three healthy lifestyle behaviors (two health promotion behaviors including physical activity, fruit and vegetable intake, as well as one disease preventive behavior) and QoL among old adults during the COVID-19 pandemic.

Socioeconomic status (SES), including educational level, professional status, and economic situation, have been demonstrated to be important predictors for physical activity, diet, preventive behaviors and QoL in the general population, respectively (20–23). For example, many studies have reported positive associations with adequate physical activity, healthy eating, and performing preventive behaviors with high economic status during the COVID-19 pandemic (16, 24–26). In addition, a recent systematic review indicated that low education levels, unemployment status, and low economic situation correlated with poorer QoL (26). However, the moderating effects of SES on the association between lifestyle behaviors and QoL among older adults are still unknown. This deserves further examination and can help to develop tailored strategies to enhance the efficacy of an intervention to improve QoL of the elderly. This can be achieved using PA, healthy diet, and preventive behaviors during the COVID-19 outbreak and future pandemics (26).

The current study aimed to (1) investigate the characteristics of QoL among Chinese older adults during the COVID-19 pandemic; (2) examine the associations of three lifestyle behaviors (physical activity, fruit and vegetable intake, and preventive behaviors) with older adults' QoL levels; (3) identify the moderating role of SES indicators (education level, professional status, and economic situation) in the associations between lifestyle behaviors and QoL levels among Chinese older adults. It was hypothesized that (1) older adults' QoL levels would differ significantly for several demographic characteristics; (2) taking up healthier lifestyle behaviors would be significantly associated with higher QoL levels among Chinese older adults; (3) specific SES indicators would significantly moderate the associations between lifestyle behaviors and QoL levels in Chinese older adults. The research may assist in understanding older adults' QoL and their potential contributors. Such information may provide useful information to inform public health and social policies focused on maintaining the overall well-being of older adults during the COVID-19 pandemic.

## Methods

### Participants

A cross-sectional study design with a convenient sampling approach was used in this study. The sample size was calculated by using G^*^Power 3.1 software with Linear Multiple Regression Fixed Model (27). For achieving a medium effect size (Cohen's *f*^2^ = 0.15) on the association between PA and QoL in previous studies in older adults (28, 29), with an alpha of 0.05, the statistical power of 80%, and a response rate of 60%, a total of 205 participants were required. Seven hundred and twenty-seven community-dwelling older adults aged 60 years and above were contacted from five cities in the Hubei province of China, including Wuhan, Xiaogan, Jingzhou, Shiyan, and Xiangyang. A total of 609 older adults (609/727, 83.8%) agreed to participate in this online survey. Participants met the eligibility criteria, including (1) aged 60 years and above; (2) not infected with COVID-19; (3) having no cognitive disorders or impairments; (4) having access to mobile phones or computers; and (5) having sufficient reading skills in Chinese. Finally, data of 516 eligible participants were included in the analysis, where 93 participants were excluded due to following reasons (1) no access to mobile phones or computer; (2) having reading disorders, and (3) repeated completion. For participants who had difficulties using mobile phones or computer operations, their family members and friends were invited to assist them in completing the online survey. The survey was conducted from 15th June 2020 to 10th July 2020, which were 3 months after the first wave of the COVID-19 pandemic in Hubei province with no lockdown restrictions in this region.

### Procedure

The online questionnaire survey was administered using an online survey platform in China, namely SOJUMP (Changsha Ranxing Information Technology Co., Ltd., China). All recruitment posters and the survey hyperlink were disseminated through mobile Short Message Service (SMS) and popular social media platforms in China such as WeChat, Weibo, and QQ. There were three approaches used for recruiting participants: (1) Relying on the researchers' social networks in five cities of Hubei province, the eligible family members, friends, and relatives of researchers were also invited. These initial participants then encouraged their friends to join the survey. (2) Researchers contacted the directors of community neighborhood committees in Wuhan and Xiaogan and sought their collaboration and support. Upon receiving the directors' agreement, researchers were permitted to enter their community neighborhood WeChat groups to recruit eligible participants. (3) Researchers contacted officials who oversaw the retirement in two universities in Wuhan. With the support of officials, a recruitment poster and survey hyperlink were delivered to their internal WeChat group, especially for retirement colleagues.

The duration of the online survey was around 15 min. Participants who completed the online survey was offered a 30 RMB incentive by electronic transfer *via* WeChat or Alipay or by prepaid telephone recharge. Participants were asked to sign an informed consent form prior to completing the questionnaire. Ethical approval for the study was obtained from the Research Ethics Committee of Hong Kong Baptist University (REC/19-20/0490).

### Measures

#### Demographic Information

Demographic characteristics included age, gender (male/female), marital status (single/married/divorced or widowed), living situation (alone/with others such as a spouse, partner or children), and three socioeconomic status (SES) related variables (26), which included educational level (primary school or below/middle or high school/college or above), professional status (unemployed/pensioner or retired/employed), and economic situation (below average/average/above average). Body weight and height were also collected for calculating the body mass index [BMI, body weight (kg)/body height squared (m^2^)]. The BMI was categorized into four levels (underweight BMI < 18.5/ healthy weight 18.5 ≤ BMI < 23/overweight 23 ≤ BMI < 26/obese BMI ≥ 26) based on previous studies for Chinese populations (30, 31).

#### Covariates

Having chronic diseases and infected cases of acquaintances were considered as health-related covariates (32, 33). Participants were asked if they had a chronic disease (e.g., heart diseases, diabetes, cancer, respiratory illnesses, liver, or kidney diseases) and if any acquaintances were (or had been) infected with COVID-19 (e.g., friends, family members, and neighbors). Answers were recorded as Yes/No.

#### Lifestyle Behaviors

Physical activity (PA) was measured using the algorithm of the stages of change for PA, adapted from a previous study (34). Participants were asked one question about PA; “Currently, do you perform at least 150 min of moderate-intensity (slightly sweating and some increase in respiration) physical activity (e.g., brisk walking, bicycling, or swimming) every week?” Answers were given on a five-point Likert-scale with “1 = No, I don't intend to start, 2 = No, but I'm considering it; 3 = No, but I seriously intend to start; 4 = Yes, but only during the outbreak of COVID-19; and 5 = Yes, this was true for a long time before the outbreak of COVID-19.” A higher score indicated a higher PA level, at which participants performed more PA.

Fruit and vegetable intake (FVI) was measured using the algorithm of the stages of change for FVI, adapted from a previous study (34). Participants were asked one question about “Currently, do you eat at least five servings of fruit and vegetable every day?” Answers were given on a five-point Likert-scale with “1 = No, I don't intend to start, 2 = No, but I'm considering it; 3 = No, but I seriously intend to start; 4 = Yes, but only during the outbreak of COVID-19; and 5 = Yes, this was true for a long period before the outbreak of COVID-19.” A higher score indicated a higher FVI level, at which participants eat more fruits and vegetables.

COVID-19 preventive behaviors include hand washing, facemask wearing, and social distancing in public areas according to the recommendations of WHO (35). A six-item structured scale was used to measure preventive behaviors, with two items for each of the three behaviors (36). In particular, the items for hand washing were “during the previous week, I adhered to washing my hands frequently with soap and water or alcohol-based hand rub (for at least 20 s, on all surfaces of the hands)” followed by two situations including “(a) in a daily life situation, e.g., before eating, and (b) in a disease-related situation, e.g., after caring for the sick.” The items for facemask wearing were “during the previous week; I adhered to wearing a face mask properly”, followed by two situations including “(a) when visiting public places, and (b) when caring for the sick.” The items for social distancing were “during the previous week, I adhered to social distancing” followed by two situations including “(a) staying out of crowded places and avoiding mass gatherings when going outside of my home, and (b) keeping space (at least 1.5 m) between myself and other people who were coughing or sneezing.” All responses were indicated on a four-point Likert scale ranging from “1 = strongly disagree” to “4 = strongly agree.” A mean score of the total six items was then computed.

#### Quality of Life (QoL)

The self-reported scale of the World Health Organization Quality of Life (WHOQOL)-BREF (37) was used to assess QoL. Two items were used from general QoL in this study based on the parsimonious principle. One item assessed the overall rating of each participant's QoL using a 5-point Likert-scale with “1 = very bad; 2 = bad; 3 = ordinary; 4 = good; 5 = very good.” The other one assessed how participants were satisfied with health using a 5-point Likert scale ranging from “1 = very dissatisfied” to “5 = very satisfied.” A mean score of two items was then calculated. The Cronbach's alpha coefficient was 0.761. In addition, the QoL was classified into three categories, including low level (mean score <3), middle level (mean score = 3), and high level (mean score >3) (38).

### Data Analysis

Data were analyzed using the IBM SPSS version 26.0. The diagnostic testing (e.g., outlier screening and distribution checking) was first conducted, and all data adhered to the normal distribution and the absolute values of skewness and kurtosis were <2. Descriptive statistics including means, standard deviation, and percentages were used to describe characteristics. *T*-tests and One-way analyses of variance (ANOVAs) tests were applied to assess the characteristics of QoL. To examine the association of PA stage, FVI stage and preventive behaviors with QoL, multiple linear regression models were used. First, the significant demographics were set as predictors entered into Model 1. Then, two covariates were added to Model 2. Subsequently, the PA stage, FVI stage, and preventive behaviors were included in Model 3.

The role of SES indicators in moderating the associations of PA stage, FVI stage, and preventive behaviors with QoL were examined using multiple linear regression analyses, respectively. Before the regression analysis, Pearson correlation analyses was used to assess the association between SES and QoL. Only SES showing significant correlation with QoL were included in the multiple linear regressions. For each multiple linear regression analysis, the significant SES were entered into Model 1. Then the significantly correlated behavior was entered into Model 2. Finally, the interaction terms between SES and significantly correlated behavior were entered into Model 3. Finally, to test the interaction terms, all the variables were mean-centered. For significant interaction terms, simple slope analyses were conducted to assess the association between QoL and behavior at low and high levels (+ 1 standard deviation) of SES. The 5% level (two-tailed) was taken as the statistical significance cutoff point.

## Results

### Characteristics of the Participants

Five hundred and sixteen eligible participants aged 60–90 years old (Mean age = 67.6 ± 6.6 yrs.) participated in the study. As shown in Table 1, the sample includes 57.9% females, and 68.6% of the participants were aged between 60 and 69 years. Most of the elderly were married (83.7%) and reported living with their spouse, partner, or children (90.7%). Nearly half (46.5%) of the old adults received college or above education, and more than half (57.9%) reported an average household income level. A total of 92.6% were pensioners/retired. 52.1% of the elderly participants were identified as overweight or obese (BMI ≥ 26 kg/m^2^). In terms of medical history, about half of the participants (50.8%) suffered from chronic diseases (e.g., heart diseases, diabetes, or cancer). A few participants reported that their acquaintances (e.g., family members, friends, or neighbors) had been confirmed with COVID-19 (9.7%). According to QoL levels, the majority of the participants (78.5%) reported high-level QoL, while 6.0% of the elderly reported low-level QoL and 15.5% of the elderly indicated middle-level QoL during the outbreak of COVID-19. The means of behaviors are shown in Table 1 [mean _PA stage_ = 3.83 (1.54); mean _FVI stage_ = 3.77 (1.49); mean _PB_ = 3.61 (0.40)].

**Table 1:** Descriptive characteristics of the study sample (*n* = 516).

**Variable**	***N*** **(%)**
**Gender**, ***n*** **(%)**	
Male	217 (42.1%)
Female	299 (57.9%)
**Living situation**, ***n*** **(%)**	
Live alone	48 (9.3%)
Live with others	468 (90.7%)
**Age group**, ***n*** **(%)**	
60–69 years old	354 (68.6%)
70–79 years old	128 (24.8%)
80 years old and above	34 (6.6%)
**Marital status**, ***n*** **(%)**	
Single	14 (2.7%)
Married	432 (83.7%)
Divorced or widowed	70 (13.6%)
**Educational level**, ***n*** **(%)**	
Primary school or below	45 (8.7%)
Middle or high school	231 (44.8%)
College or above	240 (46.5%)
**Professional status**, ***n*** **(%)**	
Unemployed	22 (4.3%)
Pensioner or retired	478 (92.6%)
Employed	16 (3.1%)
**Economic situation**, ***n*** **(%)**	
Below average	113 (21.9%)
Average	299 (57.9%)
Above average	104 (20.2%)
**Body mass index (BMI)**, ***n*** **(%)**	
BMI < 18.5 kg/m^2^	19 (3.7%)
18.5 kg/m^2^ ≤ BMI < 23 kg/m^2^	228 (44.2%)
23 kg/m^2^ ≤ BMI < 26 kg/m^2^ ≤ BMI < 26 kg/m2	206 (39.9%)
BMI ≥ 26 kg/m^2^	63 (12.2%)
**Chronic diseases**, ***n*** **(%)**	
Yes	262 (50.8%)
No	254 (49.2%)
**Infected cases of acquaintances**, ***n*** **(%)**	
Yes	50 (9.7%)
No	466 (90.3%)
**QoL, mean (SD): 3.76 (0.61)**	
Low	31 (6.0%)
Middle	80 (15.5%)
High	405 (78.5%
**Lifestyle behaviors**	
PA stage, mean (SD): 3.83 (1.54)	
FVI stage, mean (SD): 3.77 (1.49)	
Preventive behaviors, mean (SD): 3.61 (0.40)	

*SD, standard deviation; PA, physical activity; FVI, fruit and vegetable intake*.

### Characteristics of Quality of Life

As shown in Table 2, older adults' QoL differed significantly for different characteristics. There were no significant differences in QoL across gender [*t*_(514)_ = −0.26, *p* = 0.796], BMI intervals [*F*_(3, 512)_ = 1.96, *p* = 0.119], and infected cases of acquaintances [*t*_(514)_ = −1.61, *p* = 0.109]. The QoL was significantly higher for participants who had better economic situations [*t*_(2, 513)_ = 14.52, *p* < 0.001] and reported no chronic diseases [*t*_(514)_ = −5.43, *p* < 0.001]. Old adults who were married [*F*_(2, 513)_ = 5.18, *p* < 0.01] with better education [*F*_(2, 513)_ = 6.98, *p* < 0.01] reported significantly better QoL. The poorer QoL was identified among those who lived alone [*t*_(514)_ = −2.43, *p* < 0.05] and were aged over 80 years old [*F*_(2, 513)_ = 4.38, *p* < 0.05]. The employed old adults reported better QoL compared with those who were unemployed, pensioners and those who retired elderly [*F*_(2, 513)_ = 4.25, *p* < 0.05).

**Table 2:** Characteristics of quality of life (*n* = 516).

**Variable**	**QoL mean (SD)**	***F/t***	***P***
**Gender**, ***n*** **(%)**		*t*_(514)_ = −0.26	0.796
Male	3.75 (0.60)		
Female	3.77 (0.62)		
**Living situation**, ***n*** **(%)**		*t*_(514)_ = −2.43	**<0.05**
Live alone	3.51 (0.77)		
Live with others	3.78 (0.59)		
**Age group**, ***n*** **(%)**		*F*_(2, 513)_ = 4.38	**<0.05**
60–69 years old	3.80 (0.60)		
70–79 years old	3.70 (0.61)		
80 years old and above	3.51 (0.68)		
**Marital status**, ***n*** **(%)**		*F*_(2, 513)_ = 5.18	**<0.01**
Single	3.64 (0.82)		
Married	3.80 (0.57)		
Divorced or widowed	3.54 (0.74)		
**Educational level**, ***n*** **(%)**		*F*_(2, 513)_ = 6.98	**<0.01**
Primary school or below	3.44 (0.78)		
Middle or high school	3.77 (0.59)		
College or above	3.81 (0.58)		
**Professional status**, ***n*** **(%)**		*F*_(2, 513)_ = 4.25	**<0.05**
Unemployed	3.41 (0.68)		
Pensioner or retired	3.77 (0.60)		
Employed	3.90 (0.58)		
**Economic situation**, ***n*** **(%)**		*F*_(2, 513)_ = 14.52	**<0.001**
Below average	3.50 (0.68)		
Average	3.83 (0.57)		
Above average	3.86 (0.58)		
**Body mass index (BMI)**			
BMI < 18.5 kg/m^2^	3.82 (0.630)	*F*_(3, 512)_ = 1.96	0.119
18.5 kg/m^2^ ≤ BMI < 23 kg/m^2^	3.72 (0.61)		
23 kg/m^2^ ≤ BMI < 26 kg/m^2^ ≤ BMI < 26 kg/m^2^	3.84 (0.59)		
BMI ≥ 26 kg/m^2^	3.67 (0.61)		
**Chronic diseases**, ***n*** **(%)**		*t*_(514)_ = −5.43	**<0.001**
Yes	3.62 (0.61)		
No	3.90 (0.58)		
**Infected cases of acquaintances**			
Yes	3.63 (0.67)	*t*_(514)_ = −1.61	0.109
No	3.78 (0.60)		

*SD, standard deviation. Bold values denote statistical significance p-value < 0.05*.

### Association of PA Stage, FVI Stage, and Preventive Behaviors With QoL

Based on the characteristics of QoL, 6 significant demographic variables (living situation, age group, marital status, educational level, professional status, and economic situation) were entered as predictors in Model 1. Dummy variables were applied for all categorical predictors. Model 1 explained 9% of the variance in QoL (*p* < 0.001). Medical history of chronic diseases and infected cases of acquaintances were entered as covariates into Model 2 contributing to the additional explanation of 5% of the variance in QoL (Δ*R*^2^ = 0.05, *p* < 0.001). After controlling demographics and covariates, PA stage, FVI stage and preventive behaviors the lifestyle behaviors were entered to Model 3, contributing to a significant improvement in the variance explanation (Δ*R*^2^ = 0.06, *p* < 0.001). Model 3 accounted for 20% explanation power of the variance in QoL. The economic situation (*β*_average vs. below average_ = 0.17, *p* < 0.01, 95%CI = 0.08–0.33; *β*_above average vs. below average_ = 0.15, *p* < 0.01, 95%CI = 0.07–0.39), chronic diseases (*β* = 0.19, *p* < 0.001, 95%CI = 0.14–0.34), FVI stage (*β* = 0.21, *p* < 0.001, 95%CI = 0.05–0.12) and preventive behaviors (*β* = 0.10, *p* < 0.05, 95%CI = 0.03–0.29) can significantly predict the QoL of old adults. Details of regression analysis is shown in Table 3.

**Table 3:** Multiple linear regression analysis of demographics, covariate, and lifestyle behaviors with QoL (*n* = 516).

**Variable**	**Model 1**			**Model 2**			**Model 3**		
	**B (SE)**	**95%CI**	***β***	**B (SE)**	**95%CI**	***β***	**B (SE)**	**95%CI**	***β***
**BLOCK 1: DEMOGRAPHICS**									
**Living situation**									
Live alone	Reference	N/A	N/A	Reference	N/A	N/A	Reference	N/A	N/A
Live with others	0.12 (0.10)	(−0.08, 0.33)	0.06	0.13 (0.10)	(−0.07, 0.32)	0.06	0.90 (0.10)	(−0.10, 0.28)	0.04
**Age group**									
60–69 years old	Reference	N/A	N/A	Reference	N/A	N/A	Reference	N/A	N/A
70–79 years old	−0.04 (0.06)	(−0.16, 0.09)	−0.03	0.01 (0.06)	(−0.11, 0.13)	0.01	0.02 (0.06)	(−0.09, 0.14)	0.02
80 years old and above	−0.25 (0.11)	(−0.47, −0.04)	−0.10^*^	−0.20 (0.11)	(−0.41, 0.01)	−0.08	−0.13 (0.11)	(−0.34, 0.07)	−0.05
**Marital status**									
Single	Reference	N/A	N/A	Reference	N/A	N/A	Reference	N/A	N/A
Married	0.13 (0.16)	(−0.19, 0.45)	0.08	0.11 (0.16)	(−0.20, 0.42)	0.07	0.09 (0.15)	(−0.22, 0.39)	0.05
Divorced or widowed	0.06 (0.18)	(−0.29, 0.40)	0.03	0.06 (0.17)	(−0.28, 0.39)	0.03	0.04 (0.17)	(−0.29, 0.36)	0.02
**Educational level**									
Primary school or below	Reference	N/A	N/A	Reference	N/A	N/A	Reference	N/A	N/A
Middle or High school	0.16 (0.11)	(−0.05, 0.37)	0.13	0.21 (0.11)	(0.00, 0.42)	0.17^*^	0.14 (0.10)	(−0.06, 0.34)	0.12
College or above	0.13 (0.11)	(−0.09, 0.35)	0.11	0.19 (0.11)	(−0.02, 0.41)	0.16	0.11 (0.11)	(−0.10, 0.32)	0.09
**Professional status**									
Unemployed	Reference	N/A	N/A	Reference	N/A	N/A	Reference	N/A	N/A
Pensioner or retired	0.16 (0.14)	(−0.11, 0.44)	0.07	0.21 (0.14)	(−0.06, 0.48)	0.09	0.17 (0.13)	(−0.09, 0.43)	0.07
Employed	0.24 (0.21)	(−0.16, 0.65)	0.07	0.26 (0.20)	(−0.13, 0.65)	0.07	0.29 (0.19)	(−0.09, 0.67)	0.08
**Economic situation**									
Below average	Reference	N/A	N/A	Reference	N/A	N/A	Reference	N/A	N/A
Average	0.30 (0.07)	(0.16, 0.43)	0.24^***^	0.26 (0.07)	(0.13, 0.39)	0.21^***^	0.21 (0.07)	(0.08, 0.33)	0.17^**^
Above average	0.32 (0.09)	(0.16, 0.49)	0.21^***^	0.29 (0.08)	(0.12, 0.45)	0.19^***^	0.23 (0.08)	(0.07, 0.39)	0.15^**^
**BLOCK 2: COVARIATES**									
**Chronic diseases**									
Yes	–	–	–	Reference	N/A	N/A	Reference	N/A	N/A
No	–	–	–	0.26 (0.05)	(0.16, 0.36)	0.21^***^	0.24 (0.05)	(0.14 0.34)	0.19^***^
**Infected cases of acquaintances**									
Yes	–	–	–	Reference	N/A	N/A	Reference	N/A	N/A
No	–	–	–	0.12 (0.09)	(−0.05, 0.29)	0.06	0.10 (0.09)	(−0.07, 0.27)	0.05
**BLOCK 3: LIFESTYLE BEHAVIORS**									
PA stage	–	–	–	–	–	–	0.01 (0.02)	(−0.03, 0.04)	0.02
FVI stage	–	–	–	–	–	–	0.08 (0.02)	(0.05 0.12)	0.21^***^
Preventive behaviors	–	–	–	–	–	–	0.16 (0.07)	(0.03 0.29)	0.10^*^

* *Coefficient is significant at the 0.01 level*;

** *Coefficient is significant at the 0.05 level*,

*** *Coefficient is significant at the 0.0001 level*.

### Moderating Effect of Socioeconomic Status

Correlation analyses revealed that educational level (*r* = 0.13, *p* < 0.01), professional status (*r* = 0.12, *p* < 0.01), and economic situation (*r* = 0.20, *p* < 0.001) were significantly associated with QoL. In addition, except PA stage (*r* = 0.02, *p* = 0.449), FVI stage (*r* = 0.21, *p* < 0.001), and preventive behaviors (*r* = 0.10, *p* < 0.05) were significantly related to QoL.

In terms of the moderating effects of socioeconomic status between FVI and QoL, Table 4 shows that educational level, professional status, and economic situation significantly predicted old adults' QoL in model 1 (*R*^2^ = 0.22, *p* < 0.001), FVI stage significantly contributed to model 2 (Δ*R*^2^ = 0.14, *p* < 0.001), the interactions of SES with FVI stage did not significantly contribute to model 3 (Δ*R*^2^ = 0.00, *p* = 0.510). In terms of moderating effects of socioeconomic status between preventive behaviors and QoL, Table 5 shows that economic situation significantly predicted old adults' QoL in model 1 (*R*^2^ = 0.22, *p* < 0.001), preventive behaviors significantly contributed to model 2 (Δ*R*^2^ = 0.06, *p* < 0.001), the interactions of SES with preventive behaviors significantly contributed to model 3 (Δ*R*^2^ = 0.05, *p* < 0.01). In particular, 2 out of 3 interaction terms (preventive behaviors ^*^ educational level, *β* = −1.3, *p* < 0.01, 95%CI = −0.51 to −0.09; preventive behaviors ^*^ economic situation, *β* = −0.97, *p* < 0.05, 95%CI = −0.43 to −0.03) were significantly associated with QoL among old adults.

**Table 4:** Multiple linear regression examining main and interaction effects of socioeconomic status and FVI measures on QoL (*n* = 516).

**Variable**	**Model 1**			**Model 2**			**Model 3**		
	**B (SE)**	**95%CI**	***β***	**B (SE)**	**95%CI**	***β***	**B (SE)**	**95%CI**	***β***
Educational level	0.05 (0.04)	(−0.03, 0.14)	0.06	0.03 (0.04)	(−0.05, 0.11)	0.03	0.14 (0.11)	(−0.08, 0.36)	0.15
Professional status	0.17 (0.10)	(−0.03, 0.37)	0.08	0.17 (0.10)	(−0.02, 0.36)	0.08	0.28 (0.22)	(−0.15, 0.7)	0.12
Economic situation	0.16 (0.04)	(0.07, 0.24)	0.17^***^	0.14 (0.04)	(0.05, 0.22)	0.14^***^	0.07 (0.11)	(−0.14, 0.29)	0.08
FVI stage	–	–	–	0.12 (0.02)	(0.08, 0.15)	0.28^***^	0.23 (0.11)	(0.01, 0.45)	0.56^*^
FVI stage × Educational level	–	–	–	–	–	–	−0.03 (0.03)	(−0.09, 0.02)	−0.23
FVI stage × Professional status	–	–	–	–	–	–	−0.04 (0.06)	(−0.15, 0.08)	−0.18
FVI stage × Economic situation	–	–	–	–	–	–	0.02 (0.03)	(−0.04, 0.07)	0.11

*FVI, fruit and vegetable intake; B, unstandardized coefficient; SE, standard error; *β*, standardized coefficient; –, data do not include in this model*.

* *p < 0.05*;

*** *p < 0.001, 2 tailed. Model 1 R^2^ = 0.22; Model 2 R^2^ = 0.36; Model 3 R^2^ = 0.36*.

**Table 5:** Multiple linear regression examining main and interaction effects of socioeconomic status and preventive behaviors on QoL (*n* = 516).

**Variable**	**Model 1**			**Model 2**			**Model 3**		
	**B (SE)**	**95%CI**	***β***	**B (SE)**	**95%CI**	***β***	**B (SE)**	**95%CI**	***β***
Educational level	0.05 (0.04)	(−0.03, 0.14)	0.06	0.03 (0.04)	(−0.06, 0.11)	0.03	1.11 (0.39)	(0.34, 1.87)	1.16^**^
Professional status	0.17 (0.10)	(−0.03, 0.37)	0.08	0.17 (0.10)	(−0.02, 0.37)	0.08	−0.41 (0.76)	(−1.91, 1.08)	−0.18
Economic situation	0.16 (0.04)	(0.07, 0.24)	0.17^***^	0.13 (0.04)	(0.05, 0.22)	0.14^**^	0.95 (0.37)	(0.24, 1.67)	1.01^**^
Preventive behaviors	–	–	–	0.28 (0.07)	(0.15, 0.41)	0.18^***^	1.09 (0.43)	(0.24, 1.93)	0.71^*^
Preventive behaviors × Educational level	–	–	–	–	–	–	−0.30 (0.11)	(−0.51, −0.09)	−1.30^**^
Preventive behaviors × Professional status	–	–	–	–	–	–	0.16 (0.22)	(−0.27, 0.58)	0.32
Preventive behaviors × Economic situation	–	–	–	–	–	–	−0.23 (0.10)	(−0.43, −0.03)	−0.97^*^

*B, unstandardized coefficient; SE, standard error; *β*, standardized coefficient; –, data do not include in this model*.

* *p < 0.05*;

*** *p < 0.001, 2 tailed. Model 1 R^2^ = 0.22; Model 2 R^2^ = 0.28; Model 3 R^2^ = 0.33*.

**Figure 1:**
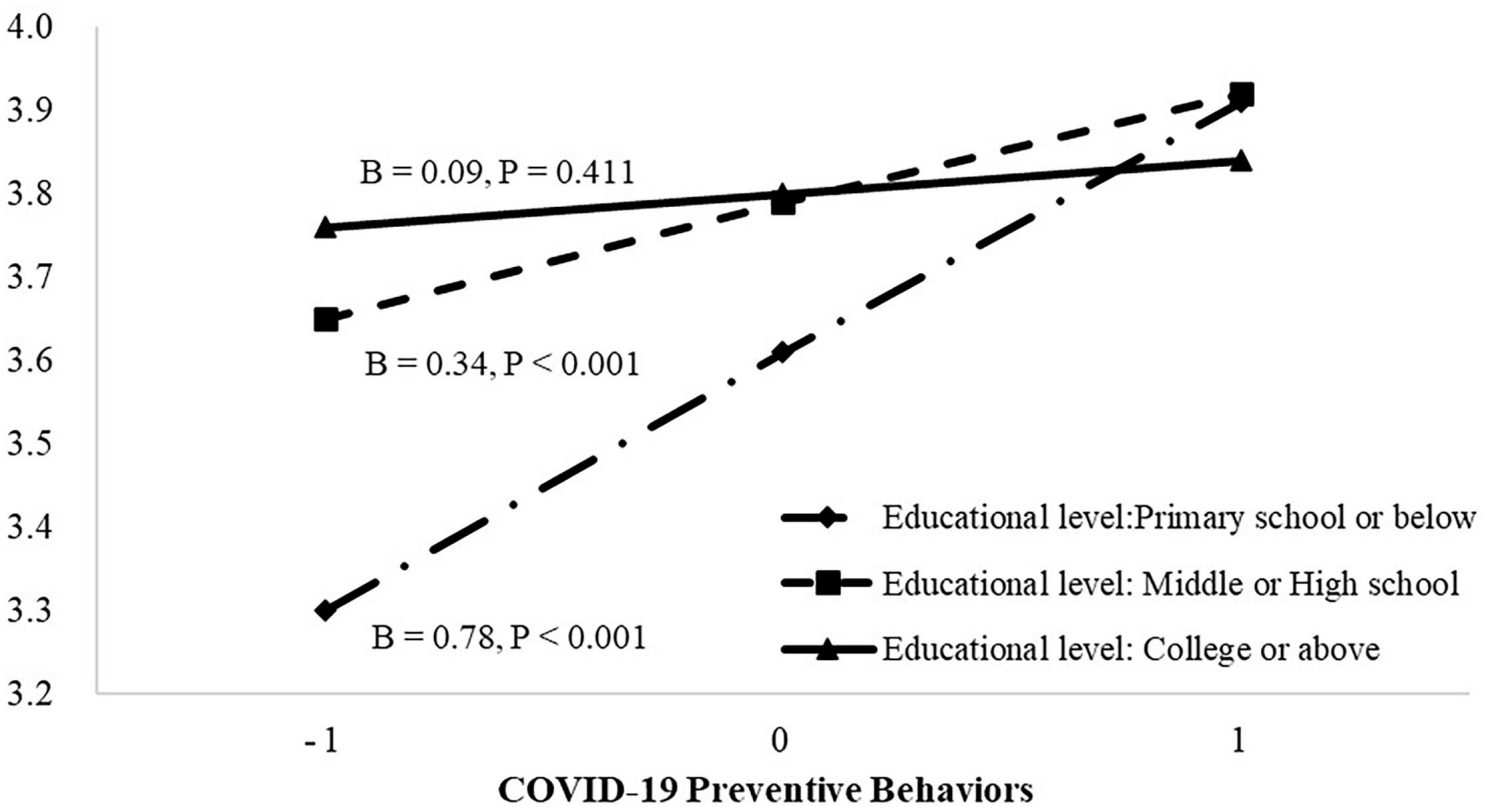
The association between COVID-19 preventive behavior and quality of life (QoL) at different categories of educational level. The plot shows the predicted values of QoL at mean and ±1 SD of preventive behavior and educational level.

**Figure 2:**
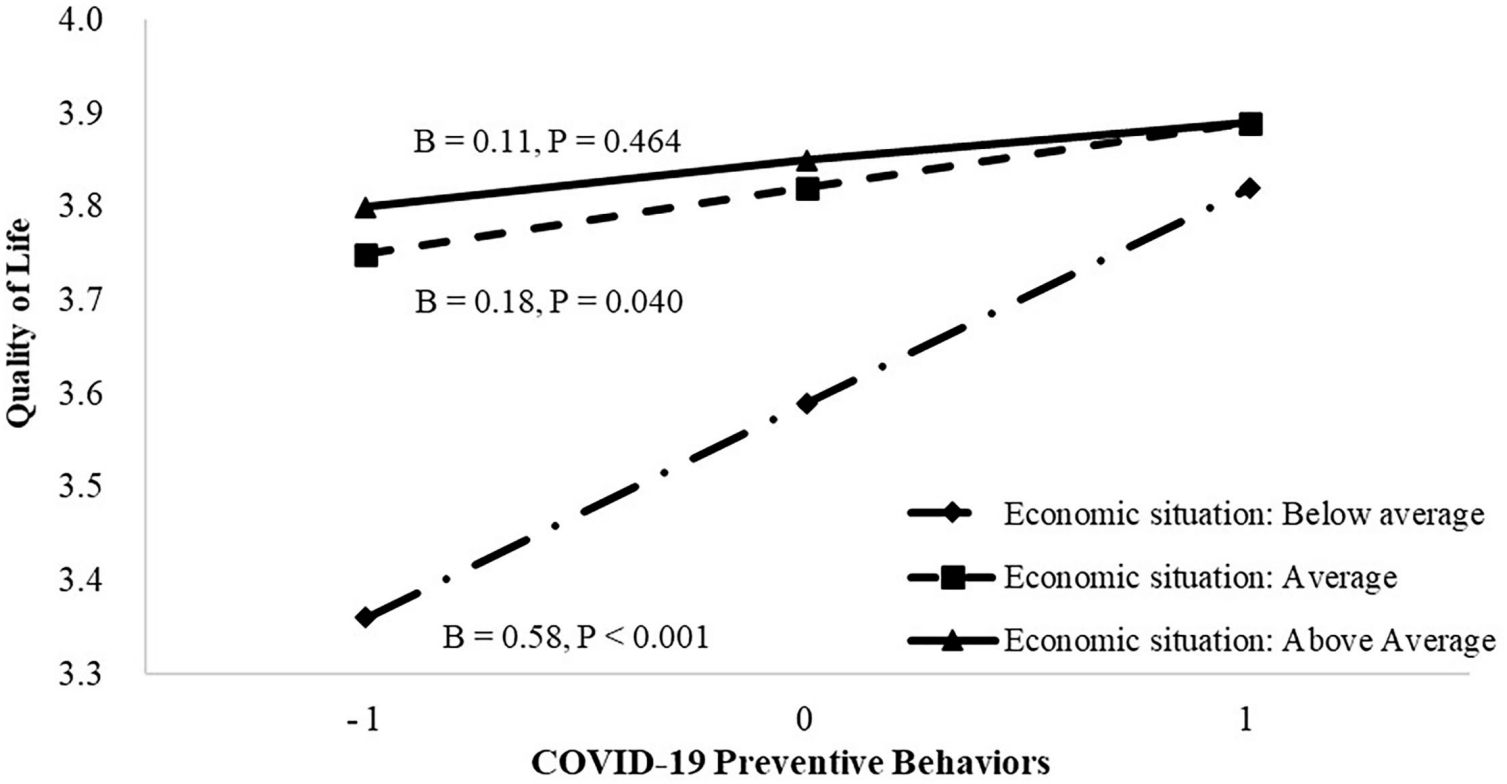
The association between COVID-19 preventive behavior and quality of life (QoL) at different categories of economic situation. The plot shows the predicted values of QoL at mean and ±1 SD of preventive behavior and economic situation.

To further analyze the significant interaction effects, simple slopes analyses was conducted. In terms of the moderating effects of education level on the relationship between preventive behaviors and QoL, Figure 1 shows that preventive behaviors were significantly associated with QoL at primary school or below of educational level [*β* = 0.78, *t*_(510)_ = 3.86, 95%CI = 0.38–1.18, *p* < 0.001] and at the middle or high school educational level [*β* = 0.34, *t*_(510)_ = 3.79, 95%CI = 0.16–0.52, *p* < 0.001], while the association was not significant at college or above for educational level [*β* = 0.09, *t*_(510)_ = 0.82, 95%CI = −0.13 to 0.32, *p* = 0.411]. In terms of the moderating effects of economic situation on the relationship between preventive behaviors and QoL, Figure 2 shows that preventive behaviors were significantly associated with QoL at the below average level for economic situation [*β* = 0.58, *t*_(510)_ = 4.46, 95%CI = 0.33–0.84, *p* < 0.001] and at the average economic situation [*β* = 0.18, *t*_(510)_ = 2.06, 95%CI = 0.01–0.36, *p* = 0.040], while the association was not significant at the above average level for economic situation [*β* = 0.11, *t*_(510)_ = 0.73, 95%CI = −0.18 to 0.41, *p* = 0.464].

## Discussion

To the best of our knowledge, this is the first online cross-sectional study to explore the characteristics of QoL, to examine the association between lifestyle behaviors and QoL, and to identify the moderating role of SES on the association between lifestyle behaviors and QoL among Chinese older adults during the COVID-19 pandemic. The findings from the study have fully supported the proposed hypotheses. Specifically, during the outbreak of COVID-19, older adults' QoL differed significantly for demographic characteristics; healthy lifestyle behaviors significantly associated with higher QoL and SES indicators such as economic situation and educational level moderated the association between lifestyle behaviors and QoL levels in Chinese older adults.

In terms of the characteristics of QoL, as suggested in previous studies, older adults with better economic situations showed higher levels of QoL than those with lower economic conditions (39, 40). In line with previous evidence, the findings revealed that the elderly with higher levels of education showed higher QoL levels (41, 42). Employed participants and the elderly below 69 years of age showed higher QoL, confirming previous research results (42). As suggested in previous studies (41), the elderly with lesser family associations demonstrated significantly poorer QoL than those with sufficient socialization. Therefore, it is not surprising that older adults who were married and lived with others (e.g., spouse, partner, or children) indicated higher QoL. Also, the older adults with chronic diseases showed a significantly poorer QoL. This finding is consistent with a recent study in Moroccan populations, which observed that the impact of COVID-19 on QoL was more marked in people with chronic health problems (43). Consistent with previous evidence, the current study did not indicate significant differences in gender and BMI (44, 45). A discrepancy with previous evidence occurred in the infected cases of acquaintances (39) where no significant differences were found in this study. This may be attributed to the reason that most of our participants reported no infected cases of acquaintances (90.3%).

In terms of the association of lifestyle behaviors with QoL, our findings were consistent with a recent cross-sectional study among polish adults (46). Older adults who were at a higher FVI stage (eating more fruits and vegetables) and adopted more individual preventive behaviors (e.g., hand washing, facemask wearing, and social distancing) were more likely to show higher QoL during the COVID-19 pandemic. Notably, the lifestyle behaviors during the COVID-19 pandemic accounted for 6% of the variance in QoL, while economic situation, SES and chronic diseases as covariates also played an important role in predicting older adults' QoL status. These findings emphasize the significance of promoting FVI and preventive behaviors during the COVID-19 pandemic among older adults. The findings also highlight the importance of considering economic and health conditions when making relevant policies and designing interventions to enhance QoL among older adults.

In terms of the moderating effects of SES indicators in the association between lifestyle behaviors and QoL, educational level, and economic situation were found to be significant moderators in preventive behaviors and QoL association. To the best of our knowledge, there are no previous studies revealing such findings. Our recent study found that economic situations could modify the relationship between COVID-19 preventive behaviors and depression among Chinese older adults (36). As depression is significantly associated with QoL in older adults (47) we infer that the moderating role of economic situation might also occur between preventive behaviors and QoL. However, more empirical research using similar study designs among older adults from other regions and countries are needed in the future. The findings of the SES moderating role in the current study revealed that when authorities motivate older adults to enact COVID-19 preventive behaviors to improve their QoL status, they need to especially focus on older adults who are at a lower economic status with lower education levels. From a social policy aspect, the findings indicate the importance and necessity of public welfare targeting socioeconomic-specific population during the pandemic prevention. For example, local government can provide relief funding and epidemic prevention appliances (e.g., face masks, disinfection alcohol, and hand sanitizer) for low-income households to facilitate their preventive behaviors (48). In addition, community administrators can organize workshops and campaigns for older adults who are at lower education levels to increase their health literacy about preventive behaviors. All these policies and measures are useful for those older adults with socio-economic disadvantages to enact more preventive behaviors, which in turn can improve their level of health-related QoL during the pandemic.

Limitations of the current study should be acknowledged. Firstly, older adults who were at low socioeconomic status may have no access to mobile phones or computers to participate in this online survey. In addition, we applied snowball sampling approach to recruit older adults from Hubei province in China. Such investigation mode and sampling method may weaken the representativeness of samples and findings. Future studies should enlarge sample size, employ randomized sampling approaches, and administrate both online and offline surveys to enhance the generalization. Secondly, all the variables were measured using self-reported subjective scales, which might lead to recall bias and social desirability effects. In addition, due to the consideration on the parsimonious mode of online survey among older adults, only two general items of QoL were addressed in this study. We acknowledge that these items were not representative enough to capture the specific domains of QoL. For PA and FVI, only the simple algorithms were used to measure the stages of change of behaviors although the validity and reliability of the questionnaire were approved in previous studies (34). Therefore, applying comprehensive questionnaires to measure QoL, PA, and FVI should be warranted in future studies. Thirdly, the socio-demographic and behavioral factors identified in the present study only explained 20% of the variance of QoL. Hence, more socio-demographics such as the number of children an older adult has, how much financial support older adults receive from their children (49) and other healthy behaviors such as restful sleep (6) should be investigated in future studies among older adults. Notwithstanding the limitations, this study provides important information on the association between lifestyle behaviors and QoL during the COVID-19 pandemic. The study also provides detail relating to the role of SES indicators in moderating lifestyle behaviors and QoL among Chinese older adults. The research findings from this study inform interventions and policy makers to improve the health and QoL of older adults by means of enhancing their lifestyle behaviors (FVI and preventive behaviors) during the COVID-19 outbreak and future pandemics.

## Conclusion

The current study investigated how Chinese older adults' demographic characteristics differ in QoL during the COVID-19 pandemic. The study also examined the association of lifestyle behaviors and QoL and identified the role of SES indicators in moderating the behavior–QoL relationship. All the study hypotheses were supported. The QoL of older adults differed significantly for living situations, age group, marital status, educational level, professional status, economic situation, and chronic diseases. The positive association of FVI and preventive behaviors with QoL was also identified in the current study. For SES indicators, only education level and economic situation significantly moderated the relationships between preventive behaviors and QoL. The research findings highlight the need for enacting preventive behaviors and FVI on enhancing QoL among older adults during the COVID-19 pandemic. The findings also revealed the importance of considering socioeconomic disparities such as economic status and education level when promoting preventive behaviors and QoL among the elderly during the pandemic. The findings presented here could be informative in implementing public health and social policies to maintain the overall well-being of older adults during the COVID-19 pandemic.

## Data Availability Statement

The raw data supporting the conclusions of this article will be made available by the authors, without undue reservation.

## Ethics Statement

The studies involving human participants were reviewed and approved by REC/19-20/0490 Hong Kong Baptist University. The patients/participants provided their written informed consent to participate in this study.

## Author Contributions

YD and WL conceived and designed the study. YD, WL, CH, and BS contributed to the preparation of study materials. YD, WL, MY, CH, and BS collected the data. MY, WL, and YD screened and analyzed the data. MY, DP, and YD drafted the manuscript. YD, JB, and WL revised and polished the manuscript. All authors have read and agreed to the published version of the manuscript.

## Funding

This research was supported by the Start-Up Grant of Hong Kong Baptist University. The funding organization had no role in the study design, study implementation, data collection, data analysis, manuscript preparation, or publication decision. This work was responsibility of the authors.

## Conflict of Interest

The authors declare that the research was conducted in the absence of any commercial or financial relationships that could be construed as a potential conflict of interest.

## Publisher's Note

All claims expressed in this article are solely those of the authors and do not necessarily represent those of their affiliated organizations, or those of the publisher, the editors and the reviewers. Any product that may be evaluated in this article, or claim that may be made by its manufacturer, is not guaranteed or endorsed by the publisher.

## Abbreviations

COVID-19: coronavirus disease 2019
PA: physical activity
FVI: fruit and vegetable intake
QoL: quality of life
SES: socioeconomic status
WHO: World Health Organization
BMI: Body Mass Index
SD: standard deviation
CI: confidence interval.
